# High frequencies of elevated alkaline phosphatase activity and rickets exist in extremely low birth weight infants despite current nutritional support

**DOI:** 10.1186/1471-2431-9-47

**Published:** 2009-07-29

**Authors:** Shannon M Mitchell, Stefanie P Rogers, Penni D Hicks, Keli M Hawthorne, Bruce R Parker, Steven A Abrams

**Affiliations:** 1 Department of Pediatrics, Baylor College of Medicine and Texas Children's Hospital, Houston, Texas, USA; 2 USDA/ARS Children's Nutrition Research Center, Houston, Texas, USA; 3 Department of Radiology, Baylor College of Medicine and Texas Children's Hospital, Houston, Texas, USA

## Abstract

**Background:**

Osteopenia and rickets are common among extremely low birth weight infants (ELBW, <1000 g birth weight) despite current practices of vitamin and mineral supplementation. Few data are available evaluating the usual course of markers of mineral status in this population. Our objectives in this study were to determine the relationship between birth weight (BW) and peak serum alkaline phosphatase activity (P-APA) in ELBW infants and evaluate our experience with the diagnosis of rickets in these infants.

**Methods:**

We evaluated all ELBW infants admitted to Texas Children's Hospital NICU in 2006 and 2007. Of 211 admissions, we excluded 98 patients who were admitted at >30 days of age or did not survive/stay for >6 weeks. Bone radiographs obtained in 32 infants were reviewed by a radiologist masked to laboratory values.

**Results:**

In this cohort of 113 infants, P-APA was found to have a significant inverse relationship with BW, gestational age and serum phosphorus. In paired comparisons, P-APA of infants <600 g (957 ± 346 IU/L, n = 20) and infants 600–800 g (808 ± 323 IU/L, n = 43) were both significantly higher than P-APA of infants 800–1000 g (615 ± 252 IU/L, n = 50), p < 0.01. Thirty-two patients had radiographic evaluation for evidence of rickets, based on P-APA greater than 800 IU/L, parenteral nutrition greater than 3 to 4 weeks, or clinical suspicion. Of these, 18 showed radiologic rickets and 14 showed osteopenia without rickets. Infants with BW <600 g were more likely to have radiologic rickets (10/20 infants) compared to those with BW 600–800 g (6/43 infants) and BW 800–1000 g (2/50 infants), p < 0.01 for each. P-APA was not significantly higher in infants with radiologic rickets (1078 ± 356 IU/L) compared to those without radiologic evidence of rickets (943 ± 346, p = 0.18).

**Conclusion:**

Elevation of P-APA >600 IU/L was very common in ELBW infants. BW was significantly inversely related to both P-APA and radiologic rickets. No single value of P-APA was related to radiological findings of rickets. Given the very high risk of osteopenia and rickets among ELBW infants, we recommend consideration of early screening and early mineral supplementation, especially among infants <600 g BW.

## Background

Metabolic bone disease is a common problem encountered in premature infants. This entity, also known as osteopenia of prematurity, often leads to morbidity in the form of fractures, which have been described in 30% of infants <1500 g birth weight (BW) [1]. Metabolic bone disease may also worsen respiratory problems in these infants [2]. In its most severe form, rickets may be present, not unlike that seen in older children. Evidence suggests that metabolic bone disease may be associated with decreased linear growth potential even after radiographic and biochemical evidence of disease is corrected [3,4].

Metabolic bone disease is characterized by decreased bone mineral density which occurs primarily as the result of decreased mineral stores in preterm infants which may be exacerbated by increased mineral demands in the neonatal period. Calcium (Ca) and phosphorus (P) are maximally acquired by the fetus during the third trimester of pregnancy, so premature infants are born with significantly lower mineral stores compared to term infants [5]. Additionally, supplementation of Ca and P in premature infants at the levels needed to match the transplacental accretion in the third trimester has proved challenging, especially in patients who do not tolerate feeds and require prolonged total parenteral nutrition (TPN) [6]. Use of medications such as corticosteroids, methylxanthines, and diuretics also appear to contribute to the development of metabolic bone disease in preterm infants [5].

Serum alkaline phosphatase activity (APA), serum P, and serum Ca have traditionally been used to screen for metabolic bone disease in preterm infants. Elevated APA and decreased serum P have been shown to correlate with increased risk of osteopenia and rickets in premature infants [7,8]. Koo et al reported correlations between the presence of skeletal demineralization and decreased birth weight, decreased gestational age, decreased enteral feeds, and elevated serum alkaline phosphatase activity levels in infants <1500 g birth weight [9]. However, the usefulness of APA and serum P as screening tools has been challenged [10]. There are currently no standard recommendations for screening of metabolic bone disease and rickets in preterm infants [11] nor are data available describing the usual values of APA or serum P in extremely low birth weight infants (ELBW, <1000 g BW).

In this study, we sought to determine usual peak serum alkaline phosphatase activity (P-APA) in ELBW infants and determine the frequency at which rickets is diagnosed in these infants.

## Methods

Using an existing database of neonatal intensive care unit (NICU) admissions, we identified all ELBW patients admitted to the Level 3 NICU at Texas Children's Hospital from January 2006 through December 2007. Their medical records were reviewed and data collected with approval from the Institution Review Board for Baylor College of Medicine and Affiliated Institutions. The following data were initially obtained: gestational age (GA), age at admission, and age at time of discharge or death. Infants excluded from further analysis were admitted to Texas Children's Hospital at age >30 days, were transferred to another institution prior to staying six weeks, or died prior to staying six weeks in the NICU.

For the remaining patients in the study, the following laboratory values were obtained: P-APA, serum Ca and serum P at the time of P-APA, and maximum conjugated bilirubin.

During this period, it was policy in our hospital to measure serum P and APA in all ELBW infants weekly until the infants were receiving full enteral feeds and had shown a clear pattern that the APA was no longer rising. Recommended practice in our hospital at that time was that a wrist or knee radiograph be obtained in infants who had a persistently high APA (usually >800 IU/L for two measurements taken at least one week apart) or had long-term inadequate mineral intake. Long term inadequate mineral intake was defined as intake below 150 mg/kg/day of calcium or 75 mg/kg/day of phosphorous for at least 3 weeks. Inadequate mineral intake was most commonly seen in infants on long term TPN (>3–4 weeks). Infants with APA <800 IU/L receiving prolonged TPN (>3–4 weeks) or who were clinically suspected to have rickets also received radiographs. All patients' radiographic records were reviewed, and patients who had been evaluated radiographically for the presence of rickets were identified. Presence or absence of the diagnoses cholestasis (defined as conjugated bilirubin >2 mg/dL), bronchopulmonary dysplasia, necrotizing enterocolitis, and gastrointestinal perforation were determined and recorded.

All radiographs of these patients which were performed for the purpose of evaluating for the presence of osteopenia and rickets were reviewed by an individual pediatric radiologist who was masked to the laboratory values associated with these patients. Radiographs obtained for these evaluations included images of the wrist(s) and/or knee(s). Patients were classified as normal, as having osteopenia (evidence of low mineral density of the bones), or as having rickets (loss of the zone of provisional calcification or metaphyseal cupping and/or fraying).

SPSS (Version 16, Chicago, IL, 2007) was used to analyze the data. General linear modeling was used to make group comparisons. Data are presented as the Mean ± Standard Deviation.

## Results

A total of 211 infants were admitted to Texas Children's Hospital NICU with a BW <1000 g between January 2006 and December 2007. Of these, 39 patients were excluded from the study because they were admitted after 30 days of age. An additional 59 patients were excluded from the study because they either died before age six weeks or were discharged before hospital length of stay reached six weeks. The remaining cohort of 113 infants was the population for which all additional data were obtained and analyzed.

In the cohort of 113 infants, the mean BW was 768 ± 153 g, and mean GA was 26 ± 2 weeks. Mean P-APA was 749 ± 326 IU/L. P-APA was found to have significant inverse relationships with BW, GA, and serum P (Table 1). Serum P had a direct correlation with BW (r = 0.36, p < 0.001).

**Table 1:** Correlations between peak alkaline phosphatase activity (P-APA) and birth weight, gestational age, and serum phosphorus.

Independent Variable	Dependent Variable	r-value	p-value
Birth Weight	P-APA	-0.36	< 0.001

Gestational Age	P-APA	-0.28	0.003

Serum Phosphorus	P-APA	-0.28	0.003

**Figure 1:**
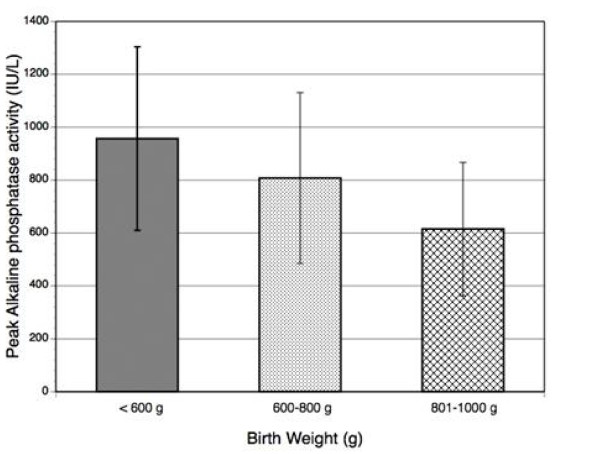
**Average peak alkaline phosphatase according to birth weight**.

In paired comparisons, P-APA of infants <600 g (957 ± 346 IU/L, n = 20) and infants 600–800 g (808 ± 323 IU/L, n = 43) were both significantly higher than P-APA of infants 800–1000 g (615 ± 252 IU/L, n = 50), p < 0.01 (Figure 1). The difference in P-APA between infants <600 g and infants 600–800 g was marginally significant (p = 0.07).

Thirty-two patients in our study population of 113 had radiographic evaluation for osteopenia or rickets. Only infants who had APA >800 IU/L on two occasions at least one week apart, who received TPN for >3–4 weeks, or who were clinically suspected to have osteopenia or rickets received these particular radiographic evaluations. Of the 32 studies, 18 showed radiologic rickets. The other 14 radiographic studies showed osteopenia without rickets. Infants with BW <600 g were significantly more likely to have radiologic rickets (10/20 infants) compared to those with BW 600–800 g (6/43 infants) and BW 800–1000 g (2/50 infants). Table 2 shows the number of infants evaluated for rickets and the number diagnosed with radiologic rickets for different ranges of P-APA.

**Table 2:** Patients evaluated for and diagnosed with rickets grouped according to peak alkaline phosphatase activity (P-APA).

P-APA (IU/L)	Number of Patients	Number Evaluated for Rickets	Number Diagnosed with Rickets
<600	42	2	1

600–800	29	6	3

800–1000	23	10	4

>1000	19	14	10

P-APA was significantly higher in infants with radiologic diagnosis of rickets (1078 ± 357 IU/L) compared to infants who were not evaluated for rickets with radiographs (642 ± 245 IU/L, p < 0.001), but not those whose radiographs did not demonstrate rickets (943 ± 346 IU/L, p = 0.18). However, four infants with rickets did have P-APA <800 IU/L. Seventeen infants with P-APA >800 IU/L did not have radiographic evaluation for rickets. Mean serum P was slightly lower in infants with rickets (5.1 ± 0.2 mg/dL) compared to infants who were not evaluated for rickets (5.6 ± 0.1 mg/dL, p = 0.03), but not those whose radiographs did not demonstrate rickets (5.3 ± 0.3 mg/dL, p = 0.52). There was no close correlation between serum Ca and the diagnosis of rickets. The BW, P-APA, and serum P for the 32 patients in which radiographic rickets evaluation were obtained are shown in Table 3.

**Table 3:** Birth weight, peak alkaline phosphatase activity (P-APA), and serum phosphorus of the 32 individuals radiographically evaluated for rickets.

Birth Weight (g)	P-APA (IU/L)	Serum Phosphorus (mg/dL)	Rickets diagnosed
437	801	5.7	Yes

460	1329	5.2	Yes

480	1013	7.4	Yes

492	474	5.3	Yes

503	799	5.7	Yes

540	760	4.9	Yes

550	1462	5.5	Yes

550	1426	3.7	No

554	1873	-	Yes

580	1334	4.6	Yes

590	839	4.3	No

590	908	4.9	Yes

611	836	4.6	No

660	1141	3.7	Yes

660	1902	6.7	No

660	1029	5.8	No

695	743	4.4	No

697	859	4	Yes

708	933	4.3	No

720	489	6.7	No

720	712	5.2	No

735	1001	6	No

740	834	6.6	No

740	1101	4.4	Yes

760	728	5.1	No

793	1083	5.3	Yes

795	863	5	Yes

795	1718	5.3	Yes

858	781	3.7	Yes

869	816	5.4	No

915	915	5.3	No

995	1107	5.6	Yes

The average maximum conjugated bilirubin of the 113 infants was 3.7 ± 5.3 mg/dL. Fifty-five of the infants had the diagnosis of cholestasis based on maximum conjugated bilirubin >2.0 mg/dL. Eighty-seven of the infants had a diagnosis of bronchopulmonary dysplasia. Twenty-five of the infants had a diagnosis of necrotizing enterocolitis, and 12 had a diagnosis of gastrointestinal perforation. Diagnoses of cholestasis, bronchopulmonary dysplasia, necrotizing enterocolitis, and gastrointestinal perforation were not significantly associated with the radiographic diagnosis of rickets. However, P-APA was higher in the 55 patients with cholestasis (867 ± 311 IU/L) compared to the 58 patients without cholestasis (636 ± 302 IU/L, p < 0.001). A total of 28/55 subjects with cholestasis (51%) had a P-APA > 800 IU/L of which 13 were > 1000 IU/L. Only 12/58 subjects without cholestasis (21%) had a P-APA > 800 IU/L of which 6 were > 1000 IU/L.

## Discussion

We found that, despite modern nutritional management, rickets is a common clinical problem in a large NICU setting. A total of 18 of 113 patients <1000 g BW were diagnosed with radiographic rickets over a 24 month time period in our NICU. Mean P-APA exceeded 600 IU/L in all subgroups and exceeded 950 IU/L in those <600 g BW. Nonetheless, among those infants who received a radiograph to evaluate rickets, there was no significant difference in P-APA in those in whom rickets was diagnosed compared to those who had osteopenia without rickets.

Although rickets is not a reportable condition in any age group in the United States, it is likely that the number of preterm infants with rickets exceeds the number of older children with rickets. Regardless, it remains clear that rickets is an important clinical event in the NICU population with substantial morbidity [1]. In addition, evidence suggests that metabolic bone disease may contribute to decreased linear growth long after biochemical and radiographic evidence of metabolic bone disease has been corrected [3,4].

The diagnosis of rickets generally remains dependent on clinical suspicion and on biochemical P-APA data with a radiograph verifying the findings. Physical examination may reveal signs of osteopenia or rickets, including pathologic fractures or bony prominences at the costochondral junctions [5]. Dual energy X-ray absorptiometry is a sensitive indicator of decreased bone mineral density which has been studied in preterm infants, but its use in the screening of osteopenia has been limited [5]. Standard radiographs currently used to evaluate infants for metabolic bone disease have limited utility because they show evidence of osteopenia only after bone density is significantly impaired [12].

We cannot identify the sensitivity as not all infants received radiographic evaluation for rickets, including seventeen infants with P-APA >800 IU/L, and therefore we cannot determine the true incidence of radiographic rickets in this population. However, we did determine that radiographic rickets developed in at least 50% of infants <600 g BW, at least 14% of infants 600–800 g BW, and at least 4% of infants 800–1000 g BW.

The reason for a high rate of rickets in infants <1000 g BW and especially those <600 g BW is unknown. In the United States, preterm formulas, fortified human milk and TPN delivered via central access provide about 80–100 mg/kg/d of retained Ca and approximately half that amount of retained P when appropriate nutrition is successfully provided. However, clinical concerns such as lack of central access, fluid restriction, and/or feeding intolerance may limit nutrient delivery, leading to suboptimal delivery of Ca and P. The *in utero *accretion rate for Ca peaks in the early 3^rd ^trimester at about 100–120 mg/kg/d [13]. The large differences in P-APA and percentage of infants diagnosed with rickets within a narrow range of birth weights may be caused by other factors in addition to differences between *in utero *Ca retention among these infants. It is possible that there is a unique and extremely elevated short term demand for minerals in these smallest infants that is not met by any of the usual nutrient delivery systems. It is also possible that elevated APA levels in these infants are not specific for the presence of rickets, and other factors including cholestasis may complicate the interpretation of elevated APA. Extremely high mineral demands in ELBW infants have not been documented in the literature, except for a single case report. This case report describes an infant with persistent radiographic rickets and elevated APA. After receiving daily calcium and phosphorus doses of 600 mg/kg and 500 mg/kg, respectively, APA levels improved [14].

Although overall growth rates are not excessive in ELBW infants, whether on TPN or enteral feeds, we suggest that very high APA and rickets may be caused by rapid bone growth in these infants which exceeds bone growth *in utero*. This rapid bone growth has not been documented in the literature. We did not document the feeding or TPN course of the infants in the study. However, acute gastrointestinal events including necrotizing enterocolitis and gastrointestinal perforation were not increased in the infants with rickets. Such events generally require reduction in enteral feeds in conjunction with increased TPN delivery.

Clinically, the total APA is often used as a marker for nutritional intervention. APA was first suggested as a screening tool for neonatal rickets based on a study including 30 premature infants [8]. Backström et al demonstrated that total APA >900 IU/L at three months corrected GA predicted radiographic osteopenia with 88% sensitivity and 71% specificity, with concomitant decreased serum P levels improving sensitivity [15]. Other studies have shown that radiographic osteopenia and rickets are correlated with APA [16,17]. P-APA has been shown to precede radiographic changes by 2–4 weeks [16]. Elevated APA is associated with hypophosphatemia [17] and decreased bone strength [18] in preterm infants. In addition, elevated P-APA has been shown to correlate with reduced neonatal growth and reduced length at 18 months [17]. However, results of a study by Faerk et al challenged the utility of APA and serum P in predicting bone mineralization in premature infants. This study demonstrated no association between bone mineral content and any of the following: P-APA, mean serum APA, and mean serum P [10].

Our data indicate that elevated APA level should not be the only factor prompting radiographic evaluation for rickets, as rickets occurred in infants even with APA <600 IU/L. Our study supports the existing data that P deficiency is important in the development of metabolic bone disease [7], but serum P is also not an adequate diagnostic tool. BW alone may be a more sensitive indicator of rickets than biochemical markers in patients with BW <600 g.

**Figure 2:**
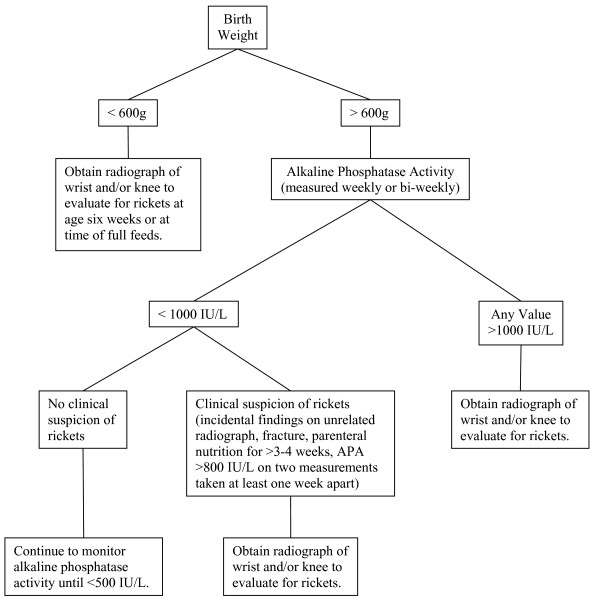
**Flow diagram to guide radiographic evaluation for rickets**.

We propose the following guidelines for clinicians evaluating ELBW infants for rickets. In general, we recommend that a radiograph of the wrist and/or knee to evaluate for rickets be obtained in premature infants when multiple measurements of APA are >800 IU/L (two levels measured at least one week apart), and/or the APA approaches or exceeds 1000 IU/L. A prolonged need for TPN (>3–4 weeks), or suspicion of fracture, or abnormal bone findings on an incidental radiograph should also trigger a radiographic evaluation for rickets. An additional policy of screening all infants <600 g BW with a radiograph of the wrist or knee when on full feeds or at about six weeks of age would also be reasonable. Infants >600 g BW with APA less than about 1000 IU/L and no clinical evidence of rickets may be followed with weekly or bi-weekly APA measurements (until levels are <500 IU/L) without additional evaluation or intervention, especially if they are receiving adequate mineral intake (Figure 2). We recommend that early nutritional intervention in infants with BW <600 g or APA >800 IU/L may be indicated to prevent a further rise in APA and the development of rickets.

## Conclusion

Elevation of APA >600 IU/L was very common in ELBW infants. BW was significantly inversely related to both P-APA and rickets. No single value of APA was related to radiological findings of rickets. Given the very high risk of osteopenia and rickets among ELBW infants, we recommend consideration of early screening and early mineral supplementation, especially among infants <600 g BW or APA >800 IU/L.

## List of abbreviations

APA: alkaline phosphatase activity; BW: birth weight; Ca: calcium; ELBW: extremely low birth weight; GA: gestational age; NICU: neonatal intensive care unit; P: phosphorus; P-APA: peak alkaline phosphatase activity; TPN: total parenteral nutrition

## Competing interests

The authors declare that they have no competing interests.

## Authors' contributions

SMM assisted in the design of the study, performed the data collection, and prepared the manuscript. SPR, PDH, and KMH participated in data collection. BRP reviewed all radiographs for osteopenia and rickets. SAA designed and coordinated the study, performed statistical analysis, and participated in preparation of the manuscript. All authors read and approved the manuscript prior to submission.

## Pre-publication history

The pre-publication history for this paper can be accessed here:


